# Writer’s Cramp

**Published:** 1886-09

**Authors:** 


### *WRITER'S CRAMP.*

Dr. de Watteville has recently published a remarkable account of a new cure of this obstinate disease, which will be good news to the large number of literary men and women, clerks, musicians, etc., who are afflicted with it. He declares that writer's cramp, or scrivener's palsy, has hitherto defied the most strenuous efforts of therapeutics. The pharmacopœia has been ransacked in the search for a suitable drug wherewith to combat the symptoms, but in vain. Variously shaped pens and supports for the hand and arm, electrical and hydropathic applications, and even protracted rest have generally proved useless in severe cases. At last, however, a system, which is described as a peculiar combination of massage and gymnastics, has been brought into operation with very remarkable success by a German, Herr Julius Wolff. This gentleman, having gained a considerable reputation in his own country, was in 1881 called to Paris by Prof. Charcot, and in two or three weeks had cured inveterate cases of writer's cramp. These cures, with others previously effected in Germany, made a considerable impression on the medical world, and when a few months ago Herr Wolff came to settle in London, his arrival was regarded with interest by many of the principal English physicians. Dr. de Watteville gives minute particulars of some of the cases in which the new treatment has succeeded in enabling sufferers from this most distressing affection to write clearly and without pain, thus confirming the observations made by the eminent Professors Billroth in Vienna, Nussbaum in Munich, and others. The massage consists of rubbing, kneading, stretching, and beating of the fingers and several muscles of the hand and arm. There are gymnastic exercises, both active and passive; and, most important of all, there are graduated exercises in writing, with a view of calling into play a new set of muscles in lieu of those injured by the cramp. The writer concludes by mentioning the case of a gentleman sent by him to Herr Wolff. Here the disease was of seventeen years'

duration, and yet in less than a fortnight, the patient was able to write for several hours a day, with almost normal rapidity and firmness.—*Midland Med. Mis.*, March 2, 1885.

